# SuAVE-Scatter: A Module for Integrating Simulations and SAXS/SANS Analyses

**DOI:** 10.1021/acs.jcim.6c00402

**Published:** 2026-04-23

**Authors:** Daniel L. Z. Caetano, Anderson A. do Espirito Santo, Diane Lima, Denys E. S. Santos, Thereza A. Soares

**Affiliations:** † Department of Chemistry, FFCLRP, University of São Paulo, Ribeirão Preto 14040-901, Brazil; ‡ Department of Physics, São Paulo State University (UNESP), Institute of Biosciences, Humanities and Exact Sciences, São José do Rio Preto 15054-000, Brazil; § Department of Fundamental Chemistry, Federal University of Pernambuco, Recife 50740-560, Brazil

## Abstract

We present two new functionalities implemented in the SuAVE software package for computing small-angle X-ray and neutron scattering (SAXS/SANS) profiles from molecular simulation data. The first routine applies the Debye scattering equation with OpenMP parallelization to calculate scattering intensities from atomic coordinates of a single structure or a trajectory file containing multiple structures. The second routine computes scattering intensities via a one-dimensional Fourier transform of atomic density profiles resolved along a user-defined axis, enabling the analysis of structured systems such as planar membranes. These approaches were validated against experimental small-angle scattering data for proteins and membranes, reproducing the data across a range of *q* values. These methods expand SuAVE analytical capabilities, enabling efficient scattering calculations for systems with diverse geometries and structural resolutions. The SuAVE_Scatter package is publicly available at https://github.com/SuAVE-Software and https://www.biomatsite.net.

## Graphical abstract

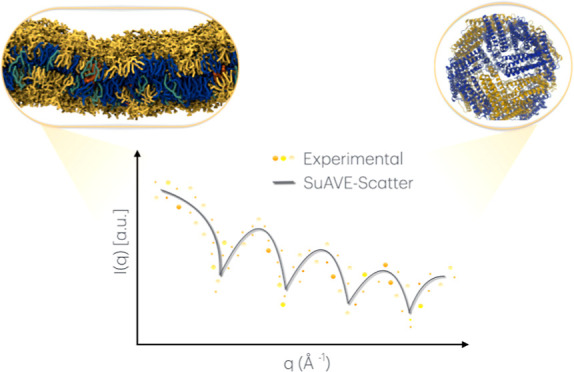

## Introduction

SuAVE (Surface Assessment via Grid Evaluation) is an open-source software package developed for the quantitative characterization of molecular surfaces and interfaces from simulation data.^1,2^ It employs a grid-based interpolation algorithm to map scalar fields such as atomic densities or order parameters onto continuous surfaces, enabling robust and efficient extraction of geometrical descriptors. A key strength of SuAVE lies in its ability to compute local and global curvature metrics directly from the reconstructed surfaces, offering detailed insight into the morphology and mechanical properties of complex interfaces. SuAVE is designed to handle chemical systems spanning a wide range of atomic densities, from soft-matter assemblies to crystalline materials, and supports both atomistic and coarse-grained representations. These capabilities make it particularly suitable for investigating phase behavior in soft-matter systems, where interfacial curvature is a key structural descriptor.

Small-angle scattering (SAS) techniques, encompassing both small-angle X-ray scattering (SAXS) and small-angle neutron scattering (SANS), have become indispensable tools for investigating the structure of materials at the nanoscale.^3–5^ These methods are particularly effective for investigating large-scale inhomogeneities and structural features in systems such as polymers, colloids, biomolecules, and porous materials.^6–9^ The capability of SAXS and SANS to nondestructively provide insights into particle size, shape, and distribution makes them invaluable for both fundamental research and industrial applications.^10–13^ Unlike higher-resolution methods such as X-ray crystallography or cryo-electron microscopy, which typically require crystalline order or extensive sample preparation, SAS enables the characterization of macromolecules in their native, solution-state ensembles. Hence, it is particularly well suited for probing systems characterized by conformational heterogeneity, including thermally driven dynamics, intrinsically flexible or disordered regions, and weakly bound or short-lived macromolecular assemblies.^14–16^

The experimental SAS data analysis is based on theoretical models that describe the intensity distributions as a function of scattering vectors. Among these models, the Debye scattering equation holds a prominent position.^17^ It allows the calculation of scattering intensities from the pair distribution function characterizing the spatial arrangement of atoms or molecules within a sample. It provides a rigorous framework for calculating the scattering intensity from polydisperse, nonperiodic, and flexible systems, as it does not require assumptions of crystallinity.^18–21^ Several programs have been successfully developed to apply the Debye scattering equation for SAXS calculations from atomic coordinates of the sample (model-to-profile approach).^22–32^ Hence, the Debye scattering equation provides an analytical framework that directly connects SAXS/SANS measurements with molecular dynamics (MD) simulations through the computation of scattering intensities from atomistic or coarse-grained models.^33,34^ This is a particularly useful approach for validating computational models against experimental SAS data and for interpreting low-resolution SAS data in the context of MD-derived structural ensembles.

However, while the Debye equation enables scattering calculations for complex morphologies, it is computationally expensive, scaling as $O\left(N^{2}\right)$ with the number of scattering centers (atoms, beads, or voxels) due to the need to evaluate all pairwise distances *r*_*ij*_. Moreover, in systems with significant thermal fluctuations or low structural order, such as lipid aggregates, Debye-based calculations can produce noisy SAXS/SANS curves unless averaged over many configurations, further increasing the computational cost. Accurate results further depend on the availability of reliable form factors or scattering lengths for all scatterers, which may not be well-defined or experimentally calibrated for coarse-grained models, leading to model-dependent uncertainty. A faster calculation of SAXS/SANS for membranes was previously proposed, which applies a one-dimensional Fourier transform to the *z*-resolved scattering density profiles, yielding form factors that can be directly compared to raw experimental measurements.^35^ Unlike the Debye-based approach, which operates directly in real space by summing pairwise contributions, the density-based approach relies on *z*-projected densities, greatly decreasing the computational cost of the calculations. While the algorithm is efficient and well-suited for lamellar systems described by one-dimensional density variations along the membrane normal, its applicability is reduced for curved or laterally heterogeneous interfaces. In SuAVE, this limitation is approached by constructing density profiles relative to a locally reconstructed interface and its surface normals, allowing interfacial curvature to be consistently accounted for in the calculation of scattering profiles.^1,2^

In this Application Note, we introduce and validate two complementary routines implemented in the SuAVE software for the calculation of SAXS and SANS observables from molecular simulations. One routine computes scattering profiles directly from atomic coordinates using the Debye equation with OpenMP parallelization,^36^ while the second derives SAXS form factors and intensities for membrane systems from one-dimensional density profiles via Fourier transformation.^35^ The SuAVE software^1,2^ is freely available at https://github.com/SuAVE-Software and https://www.biomatsite.net.

## Methodology

### Theoretical Background

We explore two established but distinct formalisms for computing small-angle scattering from molecular simulations: (i) the Debye scattering equation, which operates directly on discrete atomic coordinates, and (ii) a density-based formulation that applies a one-dimensional Fourier transform to continuous scattering-length density profiles. The following section summarizes the theoretical basis of each approach and delineates their respective domains of applicability.

### Debye Scattering Equation

We employ the well-known Debye scattering equation^9,17,27–29,37–48^ to obtain SAS profiles using SAXS or SANS

1 $$I\left(q\right)=\sum_{i=1}^{N}\sum_{j=1}^{N}f_{i}\left(q\right)f_{j}\left(q\right)\frac{\sin\left(qr_{ij}\right)}{qr_{ij}}$$

where *I* is the intensity obtained from the diffraction; *N* is the number of scatterers (in our case, atoms); q=|q⃗|=4πsin(θ)/λ is the momentum transfer vector with 2θ being the angle between scattered and incident radiation and λ the wavelength; $r_{ij}=\left|\vec{r_{i}\;}-\vec{r_{j}\;}\right|$ is the distance between atom *i* and *j*; and *f*_*i*_ is the atomic form factor of the *i*-th atom. Unless otherwise stated, the calculated intensity *I*(*q*) is reported in arbitrary units. For comparison with experiment, the theoretical profile is fitted using an overall scale factor and a constant background so that the fit reflects structural agreement in the *q*-dependent scattering features rather than trivial differences in absolute intensity normalization or background offset. Typically, we can decompose the atomic form factor into three terms, i.e.

2 $$f_{i}\left(q\right)=f_{i}^{vacuum}\left(q\right)+f_{i}^{solvent}\left(q\right)+f_{i}^{shell}\left(q\right)$$

where *f*_*i*_^vacuum^ corresponds to the intrinsic scattering of atoms in vacuum, *f*_*i*_^solvent^ is the solvent correction, and *f*_*i*_^shell^ corresponds to the solvation shell near the molecular surface. For X-ray scattering, the intrinsic atomic form factors are obtained from the fit of a 5-Gaussian analytical function.

3 $$f_{i}^{vacuum}\left(q\right)=\sum_{k=1}^{4}a_{k}\;\exp\left[-b_{k}{\left(\frac{q}{4\pi }\right)}^{2}\right]+c$$

where *a*_*k*_, *b*_*k*_, and *c* are the Cromer-Mann coefficients for a given atom.^49^ The values of the Cromer–Mann coefficients are available from the International Tables for X-ray Crystallography.^50^ The solvent affects the scattered intensity, so its contribution must be considered.^51^ A straightforward way to incorporate this effect is by adding a term proportional to the van der Waals radius of each atom, modulated by an exponential decay with respect to *q*

4 $$f_{i}^{solvent}\left(q\right)=-\rho_{bulk}V_{i}\;\exp\left(-\frac{q^{2}V_{i}^{2/3}}{4\pi }\right)$$

where ρ_bulk_ is the electron density of the solvent (for bulk water at 20 °C, ρ_bulk_ = 0.334 e/Å^3^) and *V*_*i*_ is the volume of solvent displaced by atom *i*, which is calculated using the van der Waals radius of the atom.^51^

For neutron scattering, the form factors are independent of the scattering vector and therefore can be obtained directly from tables available in the literature.^52,53^ eq 3 thus reduces to *f*_*i*_^vacuum^(*q*) = *b*_*i*_, where the *b*_*i*_ constants depend on the number of protons and neutrons that make up the atomic nucleus. Thus, isotopes of the same element, such as hydrogen and deuterium, have different neutron scattering lengths.^9,54^ To take this combination into account in the solvent composition, eq 4 becomes^55^

5 $$f_{i}^{solvent}\left(q\right)=-\eta \rho_{bulk}V_{i}\;\exp\left(-\frac{q^{2}V_{i}^{2/3}}{4\pi }\right)$$

where η = 0.1­{*b*_O_ + 2­[*b*_H_(1 – *d*) + *db*_D_]}; *b*_O_, *b*_H_, and *b*_D_ are the neutron scattering lengths of oxygen, hydrogen, and deuterium, respectively; and *d* corresponds to the deuterium concentration.

The contribution of the solvation shell is taken into account implicitly in a similar way to the CRYSOL and FoXS software.^23,27,28^ Hence, it depends on the difference between the electron density of the solvation shell in the vicinity of the solute and that of the bulk solvent (Δρ = ρ_shell_ – ρ_bulk_). The solvation shell usually has a slightly higher electron density than the bulk solvent (e.g., 10% to 20% higher).^56,57^ Therefore

6 $$f_{i}^{shell}\left(q\right)=\Delta \rho V_{i,shell}\;\exp\left(-\frac{q^{2}V_{i,shell}^{2/3}}{4\pi }\right)$$

where *V*_*i*,shell_ = *S*_*i*_Δ*w* is the hydration volume of the atom *i*, *S*_*i*_ is the solvent-accessible surface area of the *i*-th atom, and Δ*w* is the thickness of the solvation shell (typically around 3 Å).

The reduced chi-square test (χ^2^) was performed to quantitatively assess the agreement between experimental and computational scattering data for SAXS and SANS. The χ^2^ test provides a statistical measure of the goodness of fit between the experimental and theoretical data by comparing the observed scattering intensities with the corresponding calculated values, weighted by the experimental uncertainties.^6,19^ Specifically, the χ^2^ value is given by

7 $$\chi^{2}=\frac{1}{M-2}\sum_{k=1}^{M}{\left\{\frac{I^{\exp}\left(q_{k}\right)-\left[s\times I^{theo}\left(q_{k}\right)+b\right]}{\sigma \left(q_{k}\right)}\right\}}^{2}$$

where *M* is the number of experimental points; *I* ^exp^(*q*_*k*_) and *I* ^*theo*^(*q*_*k*_) are the experimental and theoretical intensities at the momentum transfer *q*_*k*_; and σ­(*q*_*k*_) is the experimental uncertainty for each individual *q*_*k*_. The reduced χ^2^ is explicitly error-weighted through the experimental uncertainties σ­(*q*_*k*_) at each *q*_*k*_ (eq 7). In this formulation, two additional adjustable parameters are included: the scale factor *s* and the background term *b*. The scale factor *s* accounts for systematic differences in the absolute scaling of intensities between experiment and theory, which can arise from uncertainties in protein concentration, detector sensitivity, or normalization procedures. The background term *b*, in turn, corrects for residual constant offsets in the experimental curve, typically due to imperfect buffer subtraction, incoherent scattering, or parasitic background contributions. Together, *s* and *b* ensure that the comparison between theoretical and experimental curves is not biased by trivial differences in scaling or baseline, allowing χ^2^ to reflect the true structural agreement.

The Debye scattering equation (eq 1) provides a general framework for computing SAS intensities from arbitrarily shaped, nonperiodic systems by explicitly accounting for all pairwise spatial correlations between scattering centers. In this formulation, the scattering intensity *I*(*q*) is evaluated directly from the Cartesian coordinates of the atoms (*x*, *y*, *z*) as a function of the magnitude of the momentum transfer vector *q*. However, the computational cost to compute eq 1 for each *q* is O­(N^2^), which limits the efficient application of the Debye scattering equation for MD simulations of large systems (*N* ≥ 100,000). This issue becomes particularly significant for soft-matter systems with continuous density distributions.

An alternative to reduce the quadratic cost of the Debye scattering equation relies on the calculation of the Fourier transform for atom density profiles. This approach applies a one-dimensional Fourier transform to real-space scattering densities from MD simulations to obtain reciprocal-space form factors.^35,58^ Experimental SAXS and SANS data are typically measured in the reciprocal space as form factors *F*(*q*_*z*_), rather than real-space density profiles. In this context, the scattering intensity for a planar membrane system (*I*(*q*) = |*F*(*q*_*z*_)|^2^) can be expressed by the following equation

8 $$I\left(q\right)={\left|\int_{-D/2}^{D/2}\left(\sum_{k}f_{k}\left(q_{z}\right)n_{k}\left(z\right)-\rho_{bulk}\right)e^{iq_{z}z}\;dz\right|}^{2}$$

Equation 8 calculates the amplitude of the structure factor in *q*_*z*_ using a one-dimensional Fourier transform of the scattering density along the *z* coordinate.^59,60^ It represents the interference of waves scattered by atoms *k*, with scattering factors *f*_*k*_(*q*_*z*_) and distributions *n*_*k*_(*z*) adjusted by the background ρ_bulk_, where *q*_*z*_ is the momentum transfer vector in the *z*-direction (normal to the membrane plane), *f*_*k*_(*q*_*z*_) is the atomic form factor of the atom *k* given by eq 3, *n*_*k*_(*z*) is the numerical density of atom *k* at position *z*, and ρ_bulk_ is the electron density of the solvent.

The routine employs the native s_dens module from SuAVE, and hence takes into account the membrane curvature for the calculation of density profiles.^1,2^ By accounting for membrane curvature and undulations using SuAVE modules, this approach eliminates the need for iterative methods to correct density distributions.^35^ We show that the resulting density profiles alone are sufficient to produce SAXS curves that closely match experimental data, provided the MD simulations are converged in time, employ system sizes appropriate for the relevant length scales, and adhere to established simulation protocols for this class of systems.

### Software Details

The computational routines were written in Fortran, and the OpenMP library^36^ was used for parallelization of the s_saxs routine. The two routines use the standard command syntax implemented in the SuAVE software.^1,2^ The flowchart for the s_saxs and s_dens_saxs modules is shown in Figure 1, where the regions highlighted in light purple and light yellow are specific to the former and latter modules, respectively. An explanatory tutorial on code usage, required files, and examples is available at https://github.com/SuAVE-Software.

**Figure 1:**
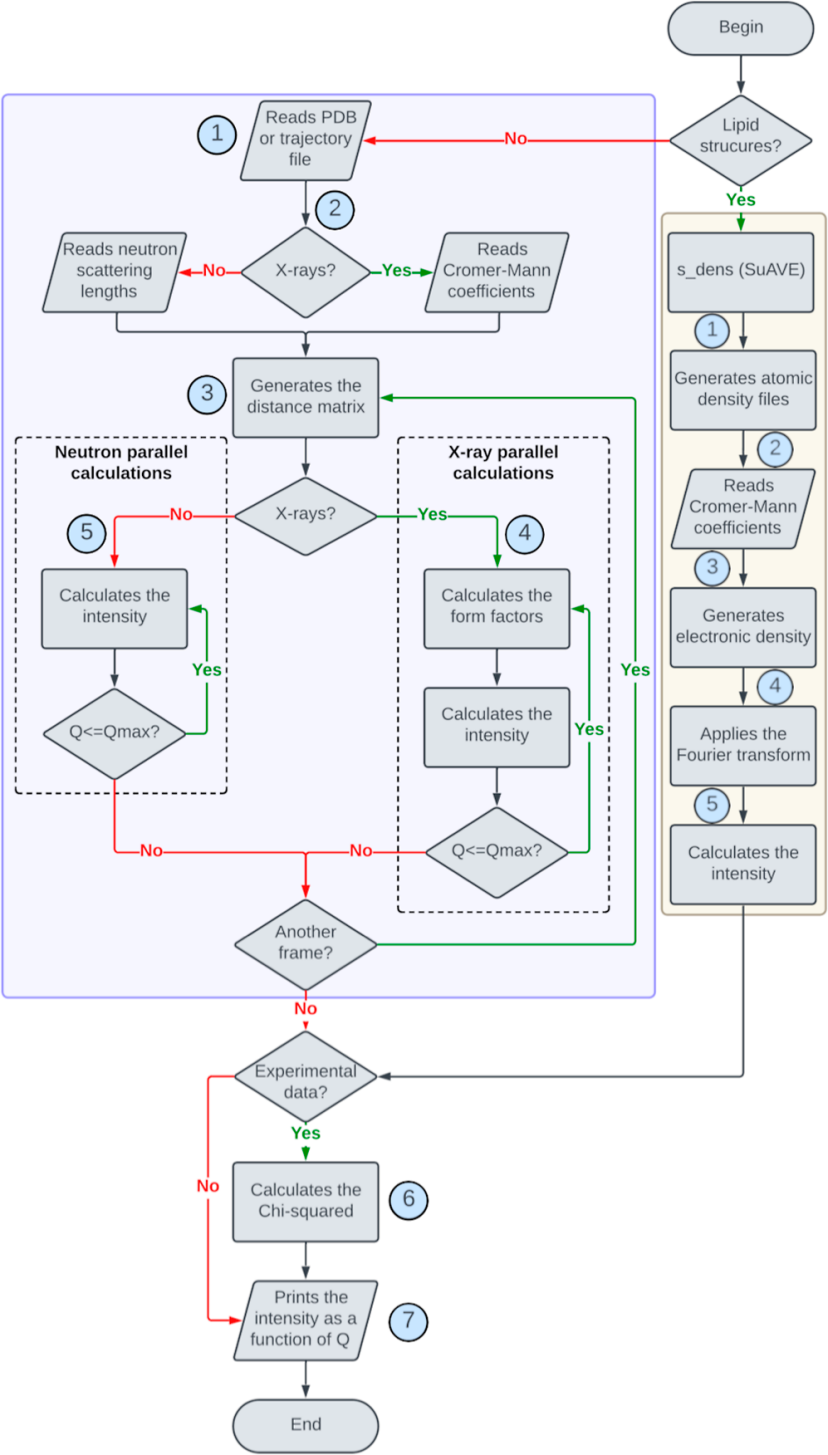
Workflow for the s_saxs (light purple) and s_dens_saxs (light yellow) routines.

The s_saxs routine operates through a sequence of steps. In Step 1, the module reads either a PDB or a trajectory file. In Step 2, the user selects the type of scattering pattern to compute. If SAXS is selected, the routine reads the Cromer–Mann coefficients; otherwise, for SANS, the neutron scattering lengths are loaded. In Step 3, the pairwise distance matrix between all atoms is generated. For SAXS calculations, the routine proceeds to compute the atomic form factors at each value of *q* (Step 4a), followed by the intensity calculation (Step 4b). This loop continues until the maximum value of *q* is reached (default parameters: *Q*_max_ = 0.5, Δ*q* = 0.001, and initial *q* = 0.001). For SANS, since form factors are *q*-independent, only the scattering intensity is computed (Step 5). If an experimental data set is provided, a χ^2^ value is calculated between the theoretical and experimental curves (Step 6). Finally, in Step 7, the intensity as a function of *q* is written to the output file. When a trajectory file is provided, Steps 3 through 6 are repeated for each frame, and the output file includes both the average intensity and its standard deviation as a function of *q*.

The s_dens_saxs routine processes atomic density profiles generated by the s_dens module of SuAVE, either from single structures or trajectory files. In Step 1, the routine generates atomic number density profiles. In Step 2, it reads the Cromer–Mann coefficients^49^ from a predefined file. Then, in Step 3, atomic number densities are converted into electron densities using the atomic form factors. In Step 4, a Fourier transform is applied to the resulting electron density profiles via eq 8, and in Step 5, the SAXS intensity profiles are computed. Although the present membrane validation focuses on SAXS, the same formalism can also be applied to SANS. As in the s_saxs routine, if experimental data are provided, a χ^2^ value is computed to quantify the agreement between theoretical and experimental curves (Step 6). Finally, in Step 7, the SAXS profiles are written to the output file, using a default *q*-range from 0.0001 to 1.0 Å^–1^. In addition to the general features described above, both routines are highly customizable, allowing the use of several optional flags (for further details, see the tutorial available at https://github.com/SuAVE-Software). These options enable, for example, the calculation of χ^2^ over a specific *q*-range (see Figure S1 in the Supporting Information).

## Results and Discussion

### The s_saxs Module

To assess the performance of the s_saxs routine in predicting SAXS profiles, we compared the calculated scattering intensities calculated with s_saxs (red lines) against those obtained using CRYSOL^23^ (green lines) and the FoXS Web server^25^ (blue lines) for six representative proteins: (A) cytochrome C (PDB ID: 1crc), (B) myosin (PDB ID: 3pn7), (C) malate synthase G (PDB ID: 1d8c), (D) Mre11-Rad50 complex (PDB ID: 3av0), (E) glucose isomerase (PDB ID: 1mnz), and (F) ferritin (PDB ID: 1ier) (see Figure 2). For consistency, the solvation-shell contribution was excluded from the profile calculations, since CRYSOL and the FoXS Web server incorporate solvation effects using different models. All atoms present in the input structures, including metal-containing or prosthetic-group moieties when present in the deposited coordinates, were retained as scattering centers in the coordinate-based calculations. The scattering intensity profiles generated by the s_saxs routine exhibit excellent agreement with those obtained from both CRYSOL and the FoXS Web server for a broad range of *q* values. To further quantify this agreement, the radius of gyration (Rg) was estimated for the six proteins using the Guinier approximation, applied uniformly within the low-*q* region defined by *q*Rg < 1.3. The resulting Rg values show remarkable agreement with the average percentage deviation between s_saxs and CRYSOL equal to 0.15%, and between s_saxs and FoXS equal to 0.46% (see Table S1 in Supporting Information).

**Figure 2:**
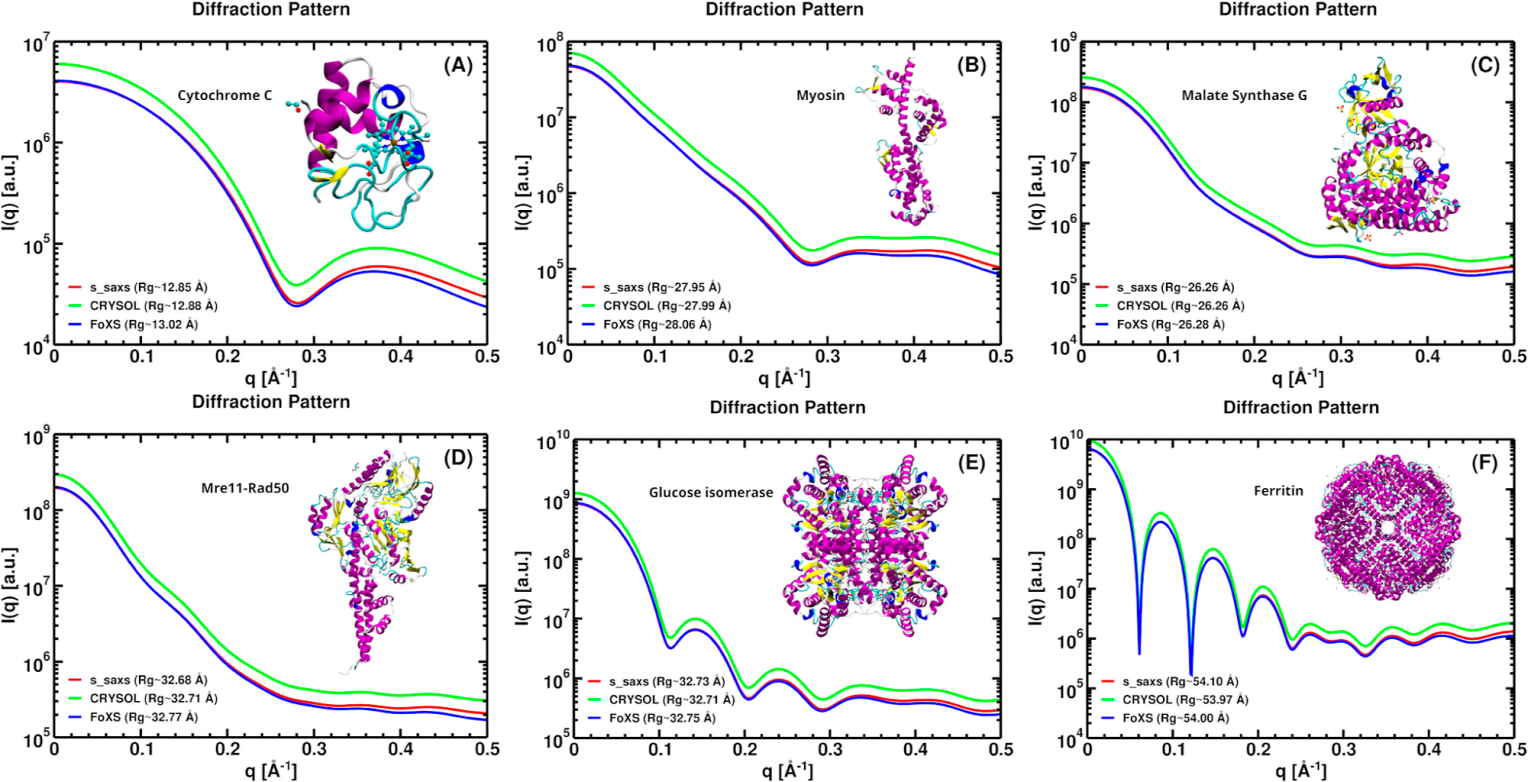
Comparison of SAXS intensity profiles for six different proteins: (A) Cytochrome C (PDB ID: 1crc), (B) Myosin (PDB ID: 3pn7), (C) Malate synthase G (PDB ID: 1d8c), (D) Mre11-Rad50 complex (PDB ID: 3av0), (E) Glucose isomerase (PDB ID: 1mnz), and (F) Ferritin (PDB ID: 1ier). The scattering intensities were calculated using s_saxs (red line), CRYSOL (green line), and the FoXS Web server (blue line). The solvation shell was not considered. As the CRYSOL and s_saxs curves overlap, for clarity, all CRYSOL curves have been scaled by a constant factor of 1.5.

The s_saxs routine calculates SAXS intensities from a single structure or from a collection of structures obtained via molecular simulations or from NMR spectroscopy, as shown for ten NMR-derived structural models of the ubiquitin protein (PDB ID: 1d3z) (Figure 3A). The s_saxs routine processes all models simultaneously, generating a single scattering intensity curve where the mean and standard deviation of *I*(*q*) are calculated for each *q* value (red line). This approach differs from both the CRYSOL and the FoXS Web server: FoXS computes *I*(*q*) for each structure separately, while CRYSOL first constructs an average structure from all models (or frames) and then computes the scattering profile from this averaged conformation. We also compared experimental and calculated SAXS profiles for lysozyme (PDB ID: 6lyz), with solvation effects treated according to each program implementation (Figure 3B). For s_saxs, the hydration-shell thickness (Δ*w* = 3 Å) and excess electron-density contrast (Δρ = 0.033 e/Å^3^) were specified through the implementation as user-adjustable inputs, rather than fixed parameters of the method. These values were chosen because they match the standard defaults commonly used in CRYSOL and FoXS and are physically consistent with a first hydration layer of about one water-molecule thickness and an electron density approximately 8–10% higher than bulk water. CRYSOL and FoXS also employ solvation models, but they rely on adjustable parameters that are optimized to fit the experimental data. All three implementations yielded good agreement with the experimental profile, with comparable χ^2^ values: 0.2006 for s_saxs, 0.2055 for CRYSOL, and 0.3632 for FoXS Web server.

**Figure 3:**
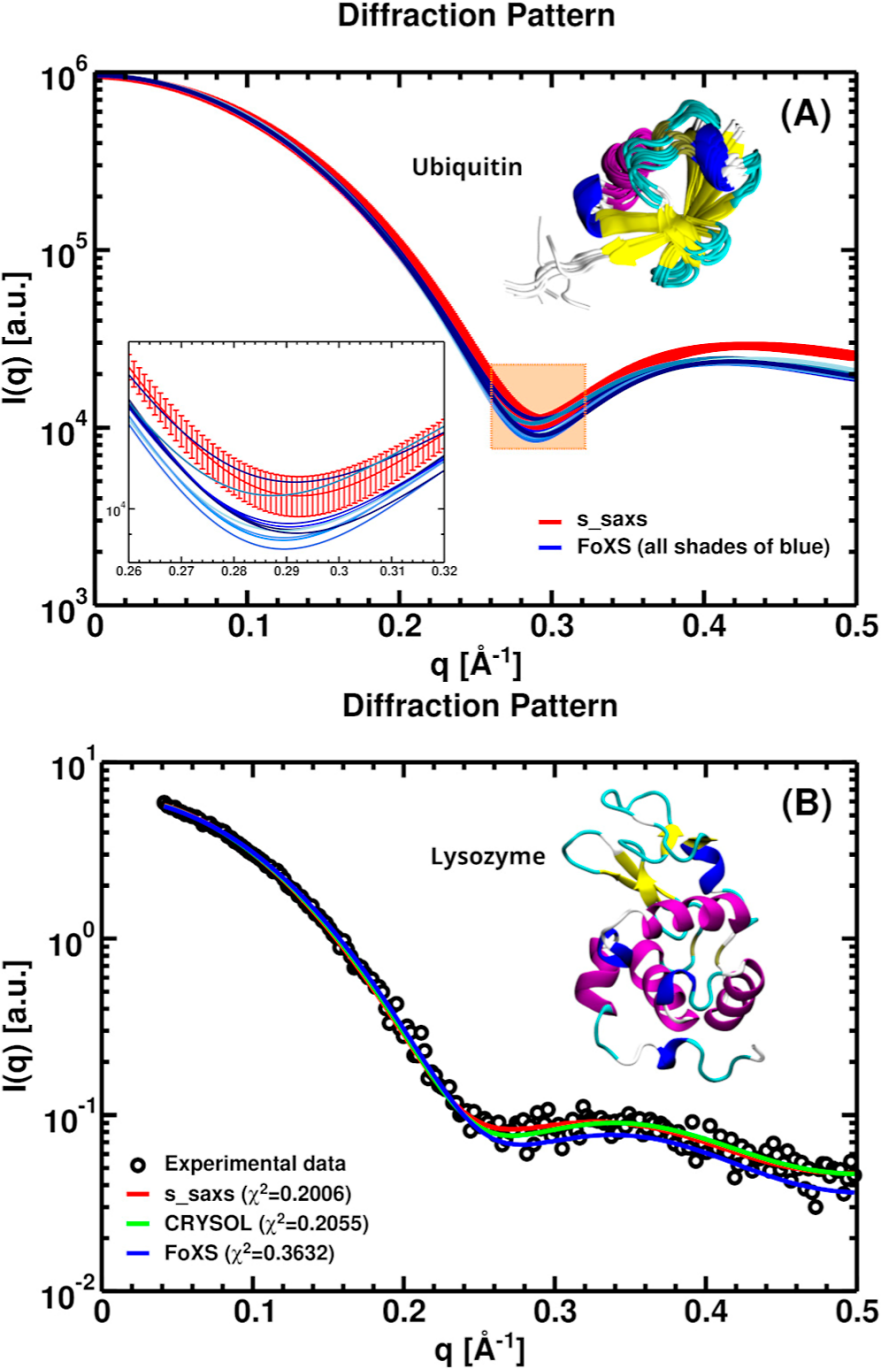
(A) Comparison of SAXS intensity profiles calculated for a set of ten NMR-derived structures of the ubiquitin protein (PDB ID: 1d3z) using s_saxs (red line) and the FoXS Web server (blue lines). The s_saxs routine computes the average *I*(*q*) and its standard deviation across the ten structures (see magnified inset). The FoXS Web server calculates *I*(*q*) separately for each structure, resulting in ten individual curves. The treatment of the solvation shell was not included in the calculations. (B) Comparison of experimental SAXS intensity (black circles) with the profiles calculated by s_saxs (red line), CRYSOL (green line), and the FoXS Web server (blue line) for the lysozyme protein (PDB ID: 6lyz). The solvation shell was calculated in accordance with the specifications of each program. For s_saxs, we used Δ*w* = 3 Å and Δρ = 0.033 e/Å^3^. Experimental SAXS data were obtained from the FoXS Web server.^25^

The s_saxs routine can be further employed to compute SANS profiles by replacing the -x (X-ray) flag with -n (neutrons). The SANS intensity profiles calculated with s_saxs and CRYSON^24^ for cytochrome C (PDB ID: 1crc), malate synthase G (PDB ID: 1d8c), and glucose isomerase (PDB ID: 1mnz) are nearly indistinguishable (Figure 4). Estimates of the radius of gyration Rg values using the Guinier approximation show excellent agreement between the two programs, with an average percentage deviation of approximately 0.15% across the three proteins and deuterium concentrations (see Table S2 in Supporting Information).

**Figure 4:**
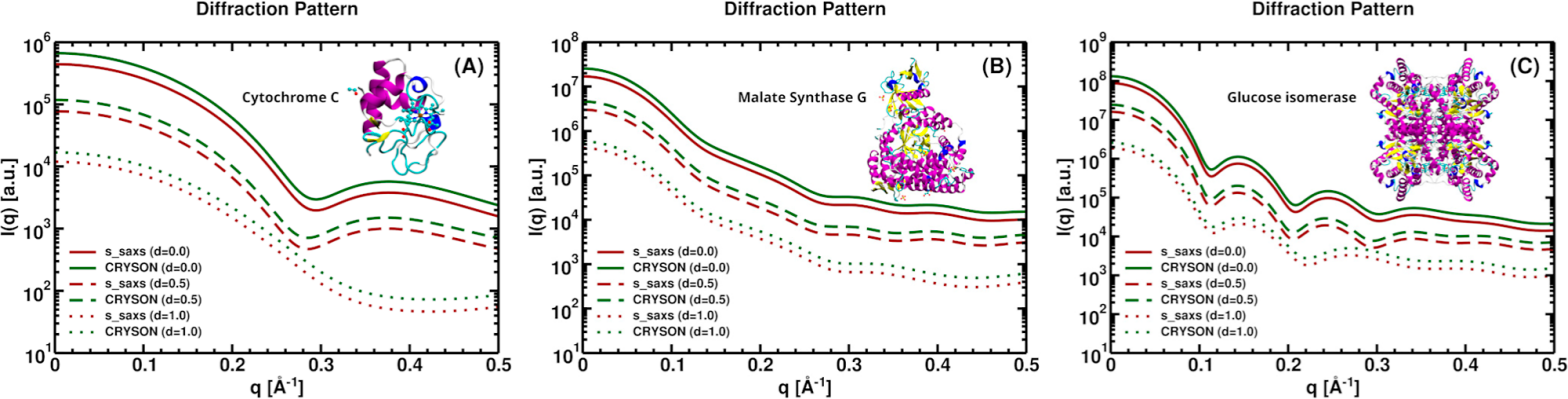
Comparison of SANS intensity profiles for the proteins (A) Cytochrome C (PDB ID: 1crc), (B) Malate synthase G (PDB ID: 1d8c), and (C) Glucose isomerase (PDB ID: 1mnz). The scattering intensity was calculated with s_saxs (dark red lines) and CRYSON (dark green lines) at three different deuterium concentrations: *d* = 0.0 (solid lines), *d* = 0.5 (dashed lines), and *d* = 1.0 (dotted lines). The solvation shell was not included in the calculations. Since the CRYSON and s_saxs curves overlap, all CRYSON curves have been scaled by a constant factor of 1.5 for clarity.

### The s_dens_saxs Module

The density-based SAXS formalism already described in the literature^35^ is suited for lamellar, quasi-planar membranes depicted by one-dimensional density profiles along the membrane normal, but inherently averages curvature and undulations, limiting sensitivity to curvature-dependent features. In SuAVE, this limitation is mitigated by the use of s_dens routine.^1^ It constructs density profiles relative to a locally reconstructed interface obtained from a grid-based representation of interfacial atoms, with densities accumulated along local surface normals to account for height fluctuations and interfacial curvature.^1,2^ Hence, the s_dens_saxs module was first validated by benchmarking SAXS profiles computed by SuAVE against those obtained with SIMtoEXP using identical density inputs (see Figure S2 in Supporting Information). Independent density profiles generated by SuAVE and SIMtoEXP yield indistinguishable SAXS profiles when processed by both codes, demonstrating the numerical consistency and correctness of the present implementation.

After establishing that our implementation quantitatively reproduces the SAXS profiles calculated with SIMtoEXP,^35^ we further validated the s_dens_saxs routine via direct comparison between calculated and experimental form factors (|*F*(*q*_*z*_)|) for four lipid membranes: (A) 1,2-dipalmitoyl-*sn*-glycero-3-phosphocholine (DPPC), (B) 1-palmitoyl-2-oleoyl-*sn*-glycero-3-phosphocholine (POPC), (C) 1-palmitoyl-2-oleoyl-*sn*-glycero-3-phospho-rac-glycerol (POPG), and (D) 1-palmitoyl-2-oleoyl-*sn*-glycero-3-phosphoethanolamine (POPE) (see Figure 5). A full description of the computational setup and simulations is presented in the Supporting Information. The experimental form factors were obtained from the NMRLipids database.^61^

**Figure 5:**
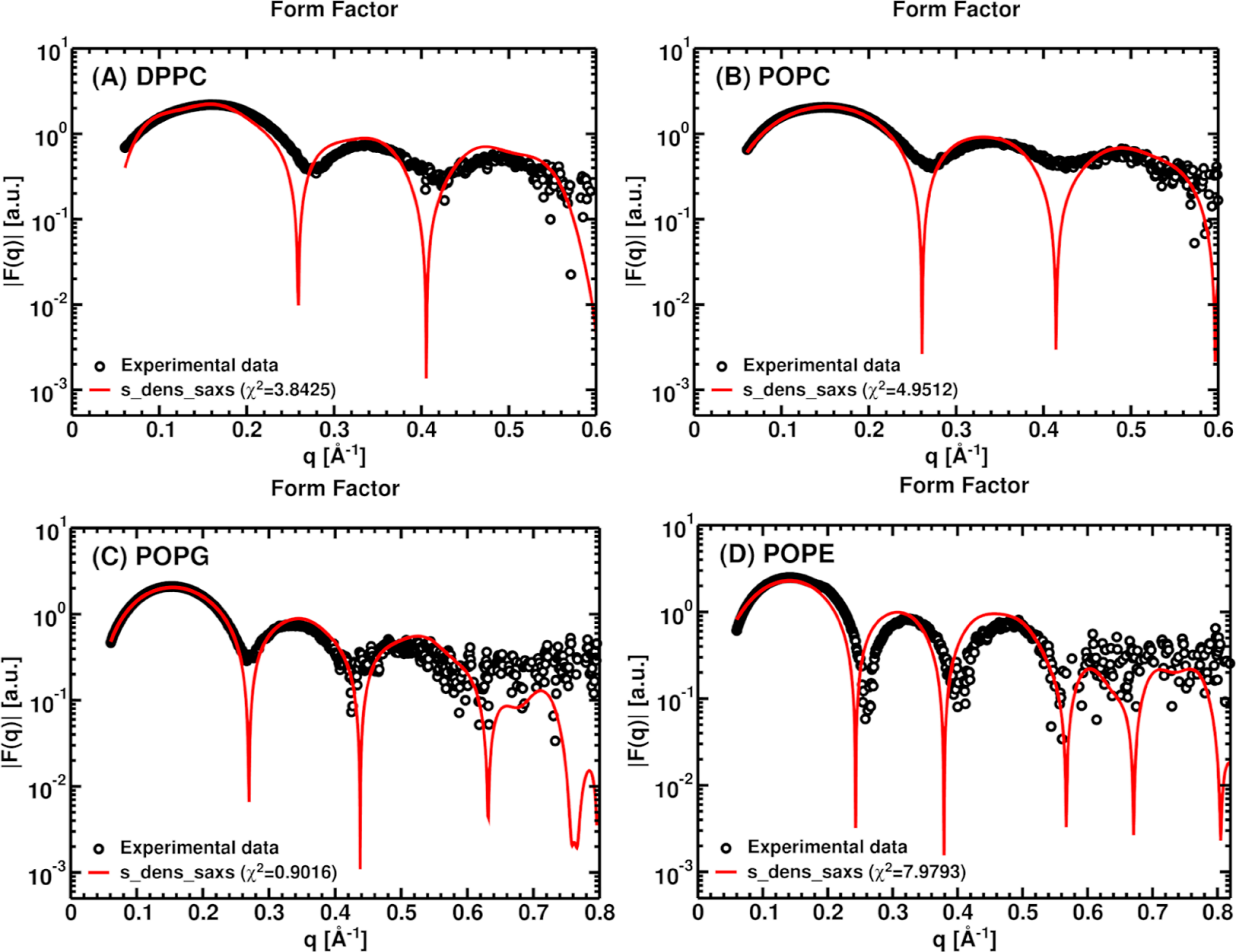
Comparison between experimental form factors (black circles) and those calculated using s_dens_saxs (red lines) for four lipid bilayers: (A) DPPC, (B) POPC, (C) POPG, and (D) POPE. The bulk density in all cases is ρ_bulk_ = 0.334 e/Å^3^. For clarity, experimental error bars are omitted; the data with error bars are provided in the Supporting Information (Figure S3).

Across all four lipid systems, the calculated form factors reproduce the overall line shape of the experimental data as well as the positions of the characteristic oscillations and minima over the investigated *q* range (see Figure 5). This level of agreement constitutes the primary validation criterion for a one-dimensional density-based Fourier formalism, as these features are predominantly determined by the bilayer thickness and the scattering-length-density contrast along the membrane normal. POPG shows the best agreement with experiment (χ^2^ = 0.90), consistent with a sharp and well-resolved scattering-length-density contrast that is accurately captured by the density-based formalism. DPPC and POPC exhibit good agreement (χ^2^ = 3.84 and χ^2^ = 4.95, respectively), but with higher χ^2^ values. It reflects scattering from the broader, dynamically hydrated phosphocholine headgroups, which smooths the interface.^62–65^ This interfacial smoothing is only partly captured by a one-dimensional density model, even though the dominant oscillation positions are preserved.

The largest deviation observed for POPE (χ^2^ = 7.98) is most consistently explained by an ensemble/force-field mismatch than by the direct computation of the SAXS form factor from the MD-derived density profile. The experimental reference points (Figure 5D) correspond to fluid-phase POPE unilamellar vesicles, for which structural parameters were obtained using the scattering density profile (SDP) approach.^66^ This approach jointly refines SAXS and contrast-varied SANS using a physically constrained real-space component model to obtain membrane structural parameters. In this framework, the area per lipid, *A*_L_, is derived from the fitted bilayer thickness and independently measured lipid volumes. MD simulations define the lipid component partitioning and their expected locations and widths along the membrane normal, providing the component distributions used to build the real-space density model that is Fourier-transformed to predict SAXS and SANS form factors.^67^

For fluid bilayers, the SAXS form factor is governed mainly by the electron-density contrast along the membrane normal. Any change in bilayer thickness or in the position and width of the headgroup and hydrocarbon density contributions shifts the oscillation minima and modulates their amplitudes. The area per lipid, *A*_L_, controls these features through packing. For a given molecular volume, a smaller *A*_L_ implies a thicker, more ordered bilayer, which redistributes electron density across the interface and alters the profile that SAXS probes. Consequently, *A*_L_ is a key structural control parameter for *F*(*q*), and deviations in the simulated packing state can translate directly into large changes in the resulting χ^2^.

This sensitivity is evident from SIMtoEXP benchmarks reported in ref 67. For POPE ULVs, SDP analysis gives *A*_L_ ≈ 58.0 Å^2^ at 35 °C. MD simulations of POPE bilayers equilibrated at a smaller area (*A*_L_ ≈ 53.2 Å^2^) show poor agreement with the SAXS form factors (χ^2^ ∼ 5.5), which improves when the same bilayer was equilibrated at higher *A*_L_ to $\approx 55.4{Å}^{2}$ (χ^2^ ∼ 3.3). Our POPE trajectories are substantially more condensed (*A*_L_ = 49.5 Å^2^ with GROMOS and 44.5 Å^2^ with CHARMM36), and further from the ULV packing state than the above-mentioned benchmark cases. The resulting χ^2^ = 7.98 therefore most naturally reflects this larger discrepancy in *A*_L_, and the associated packing/thickness bias, rather than a limitation of the form-factor calculation itself.

In summary, the s_dens_saxs module computes SAXS form factors from MD-derived density profiles by applying the one-dimensional Fourier formalism (eq 8). Comparisons against SIMtoEXP and experimental |*F*(*q*)| for DPPC, POPC, POPG, and POPE show that the implementation reproduces the expected scattering features over the relevant *q*-range. The remaining discrepancies, most evident for POPE, indicate that the agreement with experiment is mainly limited by the underlying MD ensemble (e.g., packing state and area per lipid) rather than by the form-factor calculation itself.

These bilayer results also help illustrate the broader methodological scope of the approach. Although validated here using planar lipid bilayers as a well-established test case, s_dens_saxs is more generally applicable to interfacial systems that can be described through locally resolved density variations normal to a reconstructed surface, including structured chemical or materials interfaces. This broader applicability follows directly from the SuAVE framework for the analysis of molecular surfaces and interfaces. The most natural use case of s_dens_saxs, however, remains systems whose dominant structural variation is interfacial and can be projected onto local surface normals. Systems without such a well-defined interface are better handled with the coordinate-based Debye routine.

## Supplementary Material



## Data Availability

The routines, tutorial, and data presented in this report are freely available at https://github.com/SuAVE-Software/.

## Notes

Note S1, describing the membrane systems, force field, equilibration protocol, and production MD settings for the DPPC, POPC, POPG, and POPE simulations; Table S1, reporting Guinier-derived Rg and *I*(0) values for the SAXS benchmark proteins calculated with s_saxs, CRYSOL, and FoXS; Table S2, reporting Guinier-derived Rg values for the SANS benchmark proteins at *d* = 0.0, 0.5, and 1.0 calculated with s_saxs and CRYSON; Figure S1, showing the effect of the selected *q*-range on fitting the POPC experimental form factor; Figure S2, comparing SAXS profiles computed with s_dens_saxs and SIMtoEXP using density profiles from SuAVE and SIMtoEXP; and Figure S3, comparing experimental form factors, including error bars, with calculated form factors for DPPC, POPC, POPG, and POPE (PDF)
