# Supplementary material for: SuAVE-Scatter: A Module for Integrating Simulations and SAXS/SANS Analyses
Source: J Chem Inf Model. 2026 Apr 23;66(9):5026–34. doi: 10.1021/acs.jcim.6c00402 (PMC13169381; doi:10.1021/acs.jcim.6c00402)
Supplement: Supplementary file 1 [file ci6c00402_si_001.pdf]

Supporting Information

SuAVE-Scatter: A Module for Integrating Simulations and SAXS/SANS Analyses

Daniel L. Z. Caetano*[1,2], Anderson A. do Espírito Santo[1], Diane Lima[1], Denys E. S. Santos[3], and Thereza A. Soares†[1,4]

[1]Department of Chemistry, FFCLRP, University of São Paulo, Ribeirão Preto, 14040-901, Brazil
[2]Department of Physics, São Paulo State University (UNESP), Institute of Biosciences, Humanities and Exact Sciences, São José do Rio Preto, 15054-000, Brazil
[3]Department of Fundamental Chemistry, Federal University of Pernambuco, Recife, 50740-560, Brazil
[4]Hylleraas Centre for Quantum Molecular Sciences, University of Oslo, Oslo, 0315, Norway

April 7, 2026

Contents

S1 Description of the computational setup and molecular dynamics simulations **S2**

Table S1: Guinier-derived radius of gyration and $I(0)$ of the proteins analyzed by SAXS **S3**

Table S2: Guinier-derived radius of gyration of the proteins analyzed by SANS **S3**

Figure S1: Experimental and calculated form factors for a POPC bilayer over different q-ranges **S4**

Figure S2: Comparison of SAXS profiles from `s_dens_saxs` and SIMtoEXP for a POPC bilayer **S4**

Figure S3: Comparison of experimental and calculated form factors for four different lipid bilayers **S5**

*Corresponding author – daniel.caetano@unesp.br
†Corresponding author – thereza.soares@usp.br

S1 Description of the computational setup and molecular dynamics simulations

A total of four membranes were built, composed of 384 lipid units of POPE, POPC, POPG, or DPPC. Simulations were performed with the CHARMM 36 force fields [1]. All MD simulations were performed with the GROMACS software version 2023.3 [2]. All simulations were performed for 500 ns.

- **Equilibration setup.** The protocol for building up the bilayers was separated in two stages. The initial step involves the formation of a small lipid bilayer comprising two layers of lipids arranged in an 8x4 configuration. This lipid bilayer patch was equilibrated according to the protocol outlined in Ref. [3]. The procedure included 1 ns of NVT equilibration at 200 K, followed by two stages of 25 ns of NPT simulation at 200 K and 300 K, respectively. The pre-equilibrated system was replicated along the x and y axes, resulting in a lipid bilayer containing 192 lipids per layer with approximate dimensions of 10.53 by 11.05 nm. The subsequent equilibration stage followed the same protocol as previously described. All the simulations comprising the equilibration step were performed using the leapfrog algorithm and a time step of 1 fs. Initial velocities were taken from a Maxwell distribution at each specific temperature. Bond lengths within the solute and the geometry of water molecules were constrained using the LINCS algorithm [4]. The temperatures of solute and solvent were controlled by separately coupling them to a velocity rescaling thermostat with a relaxation time of 0.5 ps [5]. The pressure was maintained at 1 bar through the Berendsen pressure coupling algorithm with a coupling constant of 0.4 ps and an isothermal compressibility of 4.5×10^{-5} bar^{-1} as appropriate for water, with semi-isotropic coordinate scaling coupling [6].

- **Production Simulations with the CHARMM force field.** After the equilibration step, simulations were performed with a 1.2 nm cutoff for long-range interactions and a time step of 2 fs. Long-range electrostatic interactions were computed using the Particle Mesh Ewald (PME) method [7], with a grid spacing of 0.16 in Fourier space. The LINCS algorithm was employed to constrain bond lengths within the solute and to maintain the geometry of water molecules [4]. Simulations of POPC, POPE, and POPG were conducted at 310 K, whereas the DPPC system was simulated at 330 K. The solute and solvent temperatures were independently regulated using a Nosé-Hoover thermostat [8, 9] with a 0.5 ps relaxation time. Pressure was kept constant at 1 bar using the Parrinello-Rahman pressure coupling algorithm [10] with a coupling constant of 5 ps, employing semi-isotropic scaling and an isothermal compressibility of 4.5×10^{-5} bar^{-1}, appropriate for water. The TIP3P water model was utilized [11], consistent with the CHARMM force field. The neighbor list was updated every 10 steps, and the system's center of mass was recentered every 100 steps.

Table S1: Guinier-derived radius of gyration and $I(0)$ values for the proteins analyzed in the SAXS section. All fits were performed using data within the $qR_g < 1.3$ range. In addition, the contributions from the solvation shell were not included in the calculations.

**PDB-ID**	s_saxs		**CRYSOL**		**FoXS Web server**	
	Rg (Å)	$I(0)$	Rg (Å)	$I(0)$	Rg (Å)	$I(0)$
1d3z	12.49	9.43×10^5	12.49	9.43×10^5	12.77	9.61×10^5
1crc	12.85	4.05×10^6	12.88	4.01×10^6	13.02	4.12×10^6
6lyz	14.35	6.20×10^6	14.35	6.20×10^6	14.49	6.31×10^6
3pn7	27.95	4.71×10^7	27.99	4.70×10^7	28.06	4.82×10^7
1d8c	26.26	1.71×10^8	26.26	1.71×10^8	26.28	1.78×10^8
3av0	32.68	1.96×10^8	32.71	1.96×10^8	32.77	2.00×10^8
1mnz	32.73	8.46×10^8	32.71	8.44×10^8	32.75	8.63×10^8
1ier	54.10	6.32×10^9	53.97	6.31×10^9	54.00	6.43×10^9

Table S2: Guinier-derived radius of gyration values (in Å) for the proteins analyzed in the SANS section. All fits were performed using data within the $qR_g < 1.3$ range. In addition, the contributions from the solvation shell were not included in the calculations.

**PDB-ID**	$d = 0.0$		$d = 0.5$		$d = 1.0$	
	s_saxs	**CRYSON**	s_saxs	**CRYSON**	s_saxs	**CRYSON**
1crc	12.69	12.69	12.76	12.77	12.28	12.19
1d8c	26.27	26.26	26.29	26.29	26.11	26.06
1mnz	32.48	32.45	32.49	32.48	31.74	31.66

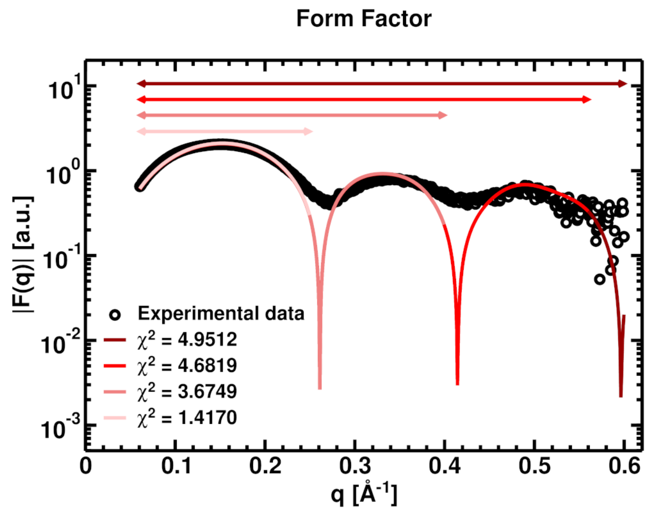

Figure S1: Comparison between the experimental form factor (black circles) and that calculated using `s_dens_saxs` for a POPC lipid bilayer over four different q-intervals: 0.0607–0.24965 Å^{-1} (light pink line), 0.0607–0.39946 Å^{-1} (pink line), 0.0607–0.55986 Å^{-1} (red line), and 0.0607–0.5998 Å^{-1} (dark red line). In all cases, the bulk electron density is $\rho_{\text{bulk}} = 0.334\,\text{e}/\text{Å}^3$. Arrows at the top of the figure indicate the q-range used in each case.

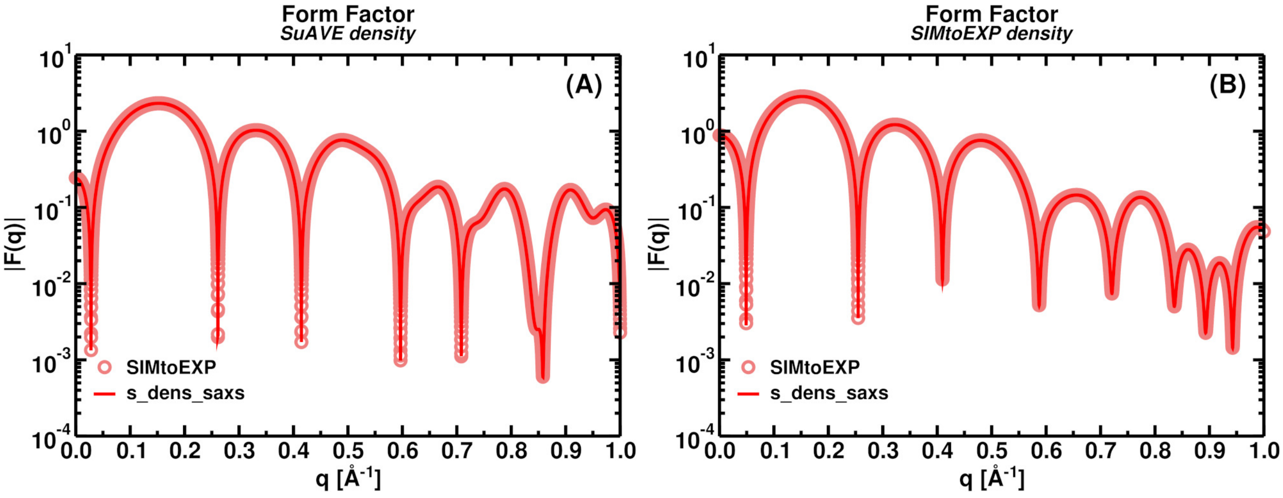

Figure S2: Comparison of SAXS profiles obtained with `s_dens_saxs` and SIMtoEXP for a POPC bilayer using two distinct numerical density distributions: (A) SuAVE and (B) SIMtoEXP. The bulk density in both cases is $\rho_{\text{bulk}} = 0.334\,\text{e}/\text{Å}^3$.

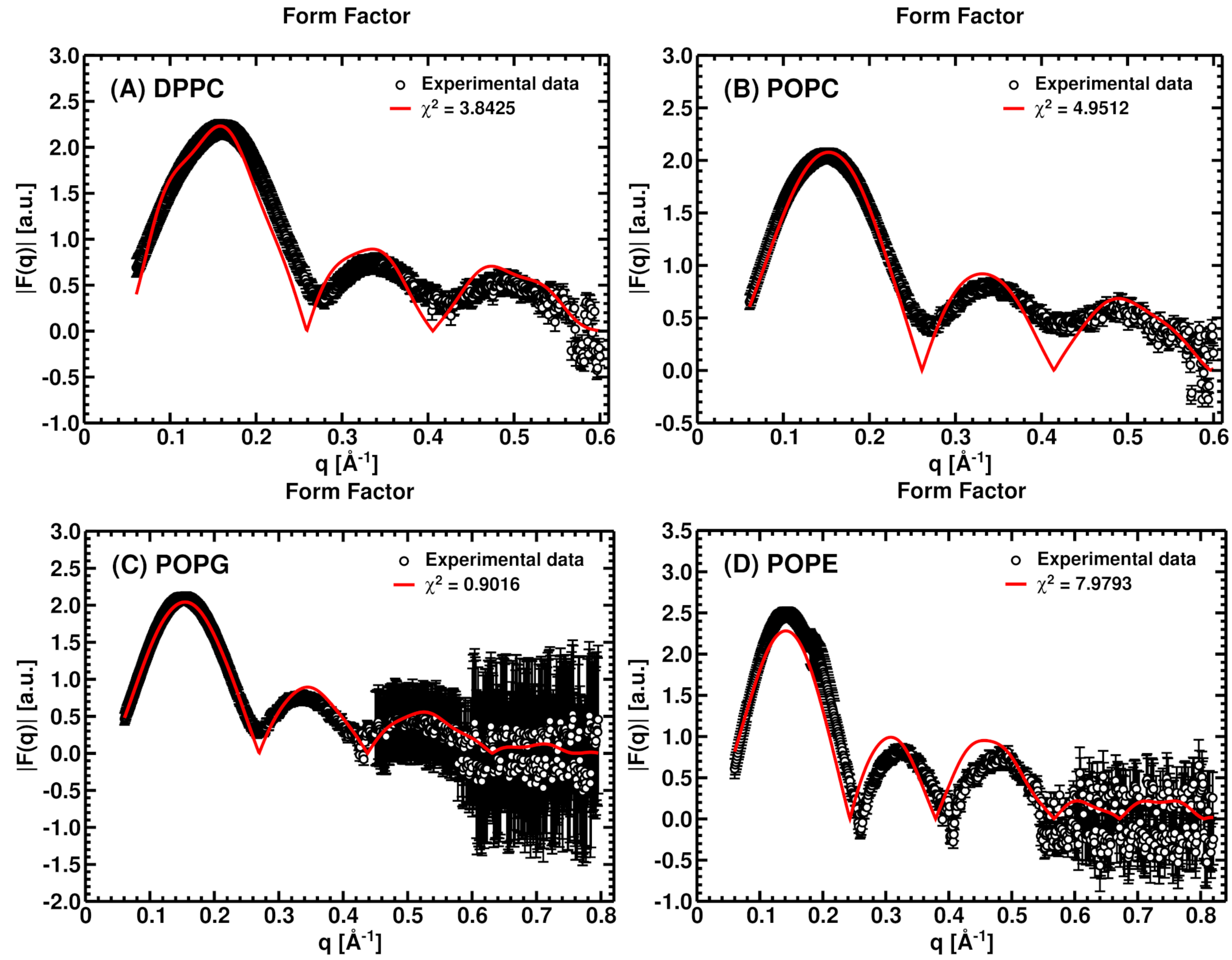

Figure S3: Comparison between experimental form factor (black circles) and those calculated using `s_dens_saxs` (red lines) for four lipid bilayers: (A) DPPC, (B) POPC, (C) POPG, and (D) POPE. The bulk density in all cases is $\rho_{\text{bulk}} = 0.334\,\text{e}/\text{Å}^3$.

References

[1] Klauda, J. B.; Venable, R. M.; Freites, J. A.; O'Connor, J. W.; Tobias, D. J.; Mondragon-Ramirez, C.; Vorobyov, I.; MacKerell Jr, A. D.; Pastor, R. W. Update of the CHARMM all-atom additive force field for lipids: validation on six lipid types. *J. Phys. Chem. B* **2010**, *114*, 7830–7843.

[2] Abraham, M.; Alekseenko, A.; Bergh, C.; Blau, C.; Briand, E.; Doijade, M.; Fleischmann, S.; Gapsys, V.; Garg, G.; Gorelov, S.; others GROMACS 2023.3 Source code. *Zenodo* **2023**,

[3] Messias, A.; Santos, D. E.; Pontes, F. J.; Lima, F. S.; Soares, T. A. Out of sight, out of mind: The effect of the equilibration protocol on the structural ensembles of charged glycolipid bilayers. *Molecules* **2020**, *25*, 5120.

[4] Hess, B.; Bekker, H.; Berendsen, H. J.; Fraaije, J. G. LINCS: A linear constraint solver for molecular simulations. *J. Comput. Chem.* **1997**, *18*, 1463–1472.

[5] Bussi, G.; Donadio, D.; Parrinello, M. Canonical sampling through velocity rescaling. *J. Chem. Phys.* **2007**, *126*.

[6] Berendsen, H. J.; Postma, J. v.; Van Gunsteren, W. F.; DiNola, A.; Haak, J. R. Molecular dynamics with coupling to an external bath. *J. Chem. Phys.* **1984**, *81*, 3684–3690.

[7] Darden, T.; York, D.; Pedersen, L.; others Particle mesh Ewald: An N log (N) method for Ewald sums in large systems. *J. Chem. Phys.* **1993**, *98*, 10089–10089.

[8] Nosé, S. U. I. A molecular dynamics method for simulations in the canonical ensemble. *Mol. Phys.* **2002**, *100*, 191–198.

[9] Hoover, W. G. Canonical dynamics: Equilibrium phase-space distributions. *Phys. Rev. A* **1985**, *31*, 1695.

[10] Parrinello, M.; Rahman, A. Polymorphic transitions in single crystals: A new molecular dynamics method. *J. Appl. Phys.* **1981**, *52*, 7182–7190.

[11] Sun, Y.; Kollman, P. A. Hydrophobic solvation of methane and nonbond parameters of the TIP3P water model. *J. Comput. Chem.* **1995**, *16*, 1164–1169.
